# In context: emotional intent and temporal immediacy of contextual descriptions modulate affective ERP components to facial expressions

**DOI:** 10.1093/scan/nsaa071

**Published:** 2020-05-21

**Authors:** Katharina M Rischer, Mattias Savallampi, Anushka Akwaththage, Nicole Salinas Thunell, Carl Lindersson, Oskar MacGregor

**Affiliations:** 1 Department of Behavioural and Cognitive Sciences, Research Institute of Health and Behaviour, University of Luxembourg, 4366 Esch-sur-Alzette, Luxembourg; 2 Department of Clinical and Experimental Medicine (IKE), Center for Social and Affective Neuroscience (CSAN), Linköping University, 581 83 Linköping, Sweden; 3 Department of Cognitive Neuroscience and Philosophy, School of Bioscience, University of Skövde, 541 28 Skövde, Sweden

**Keywords:** face processing, context, VPP, P300, LPP

## Abstract

In this study, we explored how contextual information about threat dynamics affected the electrophysiological correlates of face perception. Forty-six healthy native Swedish speakers read verbal descriptions signaling an immediate *vs* delayed intent to escalate or deescalate an interpersonal conflict. Each verbal description was followed by a face with an angry or neutral expression, for which participants rated valence and arousal. Affective ratings confirmed that the emotional intent expressed in the descriptions modulated emotional reactivity to the facial stimuli in the expected direction. The electrophysiological data showed that compared to neutral faces, angry faces resulted in enhanced early and late event-related potentials (VPP, P300 and LPP). Additionally, emotional intent and temporal immediacy modulated the VPP and P300 similarly across angry and neutral faces, suggesting that they influence early face perception independently of facial affect. By contrast, the LPP amplitude to faces revealed an interaction between facial expression and emotional intent. Deescalating descriptions eliminated the LPP differences between angry and neutral faces. Together, our results suggest that information about a person’s intentions modulates the processing of facial expressions.

## Introduction

Navigating through social environments requires efficient processing of facial expressions. For instance, faces that signal some sort of threat are detected and processed particularly quickly (Fox *et al.,* 2000; Schupp *et al.,* 2004; Mather and Knight, 2006; Krombholz *et al.,* 2007; LoBue, 2009; Van Dillen and Derks, 2012), presumably due to their high evolutionary relevance (Lang *et al.,* 1997). Electrophysiological studies suggest that faces associated with a potential threat, such as angry or fearful facial expressions, alter the amplitudes of several event-related potentials (ERPs), such as the vertex positive potential (VPP; Foti *et al.,* 2010; Luo *et al.,* 2010; Smith *et al.,* 2013), the P300 (Williams *et al.,* 2006; Luo *et al.,* 2010) and the late positive potential (LPP; Schupp *et al.,* 2004; Williams *et al.,* 2006; Moser *et al.,* 2008; Mühlberger *et al.,* 2009; Duval *et al.,* 2013).

In addition, a growing body of research suggests that the processing of facial expressions is based not only on structural facial features but on the rapid integration of these with other contextual variables, such as within-sender features (eye gaze, body posture, etc.) or external features (visual background, affective biographical knowledge, etc.) (Feldman Barrett *et al.,* 2011; Wieser and Brosch, 2012).

Several of these context–face studies suggest that contextual cues may be integrated in the processing of facial expressions as early as the VPP and (its negative counterpart) the N170 (Joyce and Rossion, 2005; Conty *et al.,* 2007, 2012; Righart and De Gelder, 2008; Hietanen and Astikainen, 2013; Diéguez-Risco *et al.,* 2015), although results for context effects on the VPP/N170 complex itself remain mixed (cf. Diéguez-Risco *et al.,* 2013) and probably vary with stimulus repetitions, task demands and the presentation form of the cues (e.g. linguistic *vs* visual; see Diéguez-Risco *et al.,* 2015; Aguado *et al.,* 2019). More reliable context effects have been found for later latencies, such as the LPP (a midline centroparietal waveform that emerges around 300–400 ms post-stimulus, sustains up to several seconds and is thought to index sustained motivated attention; Bradley, 2009; Hajcak *et al.,* 2009, 2010; Wessing *et al.,* 2013), with positive correlations between contextual threat levels and LPP amplitudes (Klein *et al.,* 2015; Xu *et al.,* 2016; Stolz *et al.,* 2019).

While most context–face studies rely on the manipulation of contextual valence to induce context effects, growing evidence suggests that other situational factors, such as temporal and spatial distancing toward (non-face) pictures with affective content, may also alter the emotional response on a behavioral and psychophysiological level (see, e.g. Simons *et al.,* 2000; Flykt *et al.,* 2007; Mühlberger *et al.,* 2008). Moreover, adopting a distant-future perspective to a recent stressor has been shown to significantly reduce reported negative affect (Yanagisawa *et al.,* 2011; Bruehlman-Senecal and Ayduk, 2015). Electrophysiological studies on delay discounting provide comparable findings: for instance, immediate relative to delayed rewards have been associated with a larger feedback-related negativity (FRN) as well as increased P300 and LPP amplitudes (Blackburn *et al.,* 2012; Cherniawsky and Holroyd, 2013; Gui *et al.,* 2016).

Although various affective within-sender and external contextual features influence the processing of faces at the electrophysiological level and non-affective contextual features such as temporal immediacy influence the processing of reward stimuli, there have to our knowledge been no previous studies on the influence of non-affective contextual features such as temporal immediacy on the electrophysiological processing of specifically facial stimuli. This study therefore aims to bridge this gap in the literature, by investigating the electrophysiological dynamics of temporal immediacy, as a part of threat-relevant contextual information presented in combination with facial stimuli.

To this end, we designed text descriptions that either escalated or deescalated the perceived threat of facial stimuli, by describing the intent of a depicted person to instigate or resolve an interpersonal conflict. In addition, for each description, one version signaled immediate intent, and the other version delayed intent. We hypothesized that (a) angry faces should result in increased ERP component amplitudes compared to neutral faces and that the effect should become apparent for relatively early ERP components, such as the VPP, and still be observable for later ERPs, such as the P300 and LPP. Moreover, we expected increased ERP amplitudes in response to (b) escalating relative to deescalating descriptions and (c) immediate relative to delayed intent. We had no clear hypothesis about (d) the onset of contextual description effects, insofar as prior research has yielded mixed results (cf. Foti and Hajcak, 2008; MacNamara *et al.,* 2009, 2010; Hietanen and Astikainen, 2013).

## Materials and methods

### Participants

Forty-six native Swedish speakers (20 identified as female; mean age ± SD: 25.04 ± 4.30 years) volunteered to participate in the study without receiving any payment. All participants reported being free of neurological or psychiatric disorders (including dyslexia), having normal or corrected-to-normal vision and being right-handed (except one ambidextrous participant), as verified by the Edinburgh Handedness Inventory—Short Form (mean score ± SD: 93.48 ± 12.28; EHI-Short Form; Veale, 2014). Four participants were excluded from ERP data analysis (two EEG data files were corrupted, which prevented their analysis; one participant had to discontinue the experiment due to time constraints; and another participant started to feel unwell, leading to early termination of the experiment). Data from 44 participants (19 of which identified as female) was thus retained for behavioral data analysis and data from 42 participants (18 of which identified as female) for ERP analysis. All participants gave their informed consent in accordance with the Declaration of Helsinki prior to participation. The Regional Ethical Review Board in Gothenburg approved the study.

### Stimulus material

We selected pictures of 32 models (16 female, 16 male) from the Umeå University Database of Facial Expressions (Samuelsson *et al.,* 2012), showing each model with a neutral and an angry (threatening) face in frontal view, for a total of 64 faces. Models were selected based on normative accuracy ratings for neutral and angry faces (mean percentage ± SD: 95.61 ± 1.24; Samuelsson *et al.,* 2012). Each picture (500 x 750 pixels) was displayed centrally in color on a 23 inch screen (HP Z23i IPS Display) with a 1920 × 1080 pixel resolution, on a dark gray background.

We derived our contextual descriptions from an online pilot study. Eighteen native Swedish speakers (10 identified as female; mean age ± SD: 32.94 ± 11.54) rated Swedish descriptions on 9-point Likert scales with regard to valence (very negative = 1 to very positive = 9) and arousal (not at all arousing = 1 to very arousing = 9) in PsyToolkit (Stoet, 2017). Thirty of these descriptions signaled escalating emotional intent (e.g. ‘She/he will beat somebody’), and 30 signaled deescalating emotional intent (e.g. ‘She/he will forgive somebody’). Additionally, we manipulated the immediacy of the intended action by creating two versions of each description, one signaling immediate intent (e.g. ‘She/he is about to yell at somebody’) and one delayed intent (e.g. ‘She/he will yell at somebody later’), for a total of 120 descriptions rated. Based on the valence and arousal ratings, we selected 16 escalating and 16 deescalating contextual descriptions, each with an immediate-intent and delayed-intent variant, for a total of 64 unique descriptions (the complete statistical results are reported in the Supplementary Materials).

We created four sets of stimuli by combining eight escalating descriptions with eight angry faces, and the remaining eight escalating descriptions with eight neutral faces. We then repeated the procedure for the deescalating descriptions and other (angry and neutral) faces. In order to control for any effects of intrinsic facial features, we created two versions of the task. Descriptions that were combined with angry faces in one version were combined with neutral faces of the same models in the other version, and vice versa, such that no models occurred as both angry and neutral in the same version. The two versions were counterbalanced across participants.

Each description was presented twice per set (both times with the same face), once with immediate intent and once with delayed intent, resulting in 16 trials per set (e.g. eight escalating angry face immediate intent and eight escalating-angry-face-immediate-intent trials in one set). The four sets were then mixed into blocks of 64 trials, with the trials presented in a pseudorandomized order so that no trial of the same set was followed by another trial of the same set more than twice in a row. The face–description pairings of the sets were held constant between blocks. In total, participants completed five blocks each, such that each of the eight available conditions (angry *vs* neutral face x escalating *vs* deescalating description x immediate vs. delayed intent) was viewed 40 times in total. The participants took short breaks between each block.

### Procedure

After giving informed consent, participants were seated in a sound-attenuated experimental room, EEG sensors were attached, and they received verbal instructions. Participants were told that they would be viewing pictures of faces with different expressions preceded by descriptions. They were instructed to attentively read and view the presented stimuli and to subsequently rate each picture in terms of valence and arousal on two 9-point Likert scales (1–9, as for the pilot study) by entering the respective numbers on a keyboard.

**Fig. 1:**
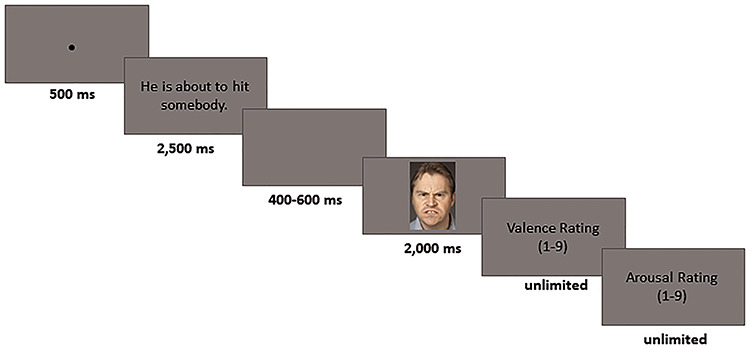
Schematic experimental run. Participants were asked to read descriptions, differing in emotional intent and temporal immediacy followed by the presentation of an angry or neutral face. After each trial, valence and arousal ratings were obtained on 9-point Likert scales. Facial expressions were taken from the Umeå University Database of Facial Expressions (Samuelsson et al., 2012). Figure reprinted with permission.

Participants viewed the stimuli at approximately 100 cm distance from the screen. Each trial started with a fixation cross presented for 500 ms, followed by the description for 2500 ms. After a subsequent interval (a blank screen), with a randomized jittered latency of 400 to 600 ms, a face was shown for 2000 ms. Following each trial, participants rated the pictures without time limit. Presentation of the stimuli was controlled by E-Prime 2.0 software (Psychology Software Tools). The complete experiment took approximately 90 min per participant. Figure 1 presents a schematic experimental run.

### E‌EG recording and data reduction

Brain activity was recorded using 33 active Ag/AgCl electrodes (AF3/4, Fz/3/4/5/6/7/8, FCz, T7/8, Cz/3/4, CPz/1/2, Pz/3/4, POz/3/4, Oz/1/2) (g.LADYbird electrodes, manufactured by g.tec), positioned according to the International 10/20 placement system on a stretchable cap (g.GAMMAcap). Two electrodes were also attached to the left and right mastoids for later offline re-referencing. Ocular movements were captured by attaching electrodes supra- and suborbit of the right eye and at the external canthi of each eye. The active electrode impedances were transformed by the system to output impedances of about 1kOhm. Electrodes were online referenced to Cz, while an additional electrode at AFz served as ground. Data was acquired in MATLAB R2017a (MathWorks) with the g.USBamp (g.tec) amplifier. It was band-pass filtered online on an internal digital signal processor within the amplifier, using a sixth-order Butterworth filter with a half-power (−3 dB) cutoff at 0.01 and 100 Hz, and sampled at 512 Hz.

Offline analysis was performed using the toolboxes EEGLAB 14.1.1 (Delorme and Makeig, 2004) and ERPLAB 6.14 (Lopez-Calderon and Luck, 2014) in MATLAB R2017a. Continuous EEG data was downsampled from 512 to 256 Hz to facilitate quicker analysis and re-referenced to the average of the mastoids, as both the VPP and the LPP are particularly pronounced for the average mastoid reference (Joyce and Rossion, 2005; Hajcak *et al.,* 2012; Rossion and Jacques, 2012), and an average ‘whole-head’ reference is not recommended for the relatively small number of channels we used in this experiment (Luck, 2014).

As a preprocessing step for removing artifacts with independent component analysis (ICA), the EEG was filtered offline using a windowed sinc FIR filter with a half-amplitude (−6 dB) cutoff at 1 (1651 points) and 40 Hz (167 points) (Winkler *et al.,* 2015). Bad channels were rejected using the joint probability of the electrode (channels containing activity three SD above probability activity limits were rejected), and the EEG data was segmented into epochs beginning 400 ms prior to picture onset and continuing until picture offset (after 2000 ms). Epochs exceeding a threshold of ±500 μV were automatically rejected, and Infomax ICA was run in EEGLAB 14.1.1.

Subsequently, ICA weights (together with their sphere matrices) were transferred to a data set that underwent the same preprocessing steps as the ICA-processed data set, except that it was filtered offline using a windowed sinc FIR filter with a half-amplitude (−6 dB) cutoff at 0.1 (8448 points) and 30 Hz (114 points), in order to avoid the potential attenuation of slow wave potentials. Multiple Artifact Rejection Algorithm (MARA; 2013) was then run to identify and remove ICA components reflecting artifacts (Winkler *et al.,* 2011). Following this, data from previously rejected channels was interpolated using superfast spherical interpolation in EEGLAB 14.1.1, and stepwise artifact rejection was performed in ERPLAB 6.14 (epochs containing step-like activity greater than 30 μV in a moving window of 400 ms with a step size of 10 ms were rejected), resulting in an overall rejection rate of 8.10% of all epochs (for a detailed overview, see Tables 1 and 2). Subsequently, epochs were averaged for each participant and each experimental condition.

**Table 1:** Percentage of rejected epochs and remaining average number of epochs included in the statistical analyses

Condition	Percentage of rejected epochs (%)	Average number of epochs
Angry face—escalating description—immediate intent	7.14	37.14
Angry face—escalating description—delayed intent	8.21	36.71
Angry face—deescalating description—immediate intent	7.92	36.83
Angry face—deescalating description—delayed intent	10.24	35.90
Neutral face—escalating description—immediate intent	7.14	37.14
Neutral face—escalating description—delayed intent	7.74	36.90
Neutral face—deescalating description—immediate intent	7.98	36.81
Neutral face—deescalating description—delayed intent	8.39	36.64

Note: Five of our datasets were subjected to an additional automatic epoch rejection procedure in EEGLAB called “automatic artifact rejection”, which is based on the iterative rejection of epochs outside five SD, with a maximum of eight iterations, prior to running ICA.

**Table 2:** Interpolated analyzed channels

Channel	Number of channel interpolations (of total *N* = 42)
Cz	4
CP1	3
CP2	1
CPz	1
Pz	3

All ERP components were quantified on the basis of their mean amplitude within specific time windows and at specific recording sites, selected based on reports of previous research, as detailed below. The VPP was analyzed at the vertex (Cz) from 120 to 180 ms after picture onset (cf. Pourtois *et al.,* 2000; Rossion and Jacques, 2012). The P300 was analyzed over the parietal midline (Pz) from 250 to 400 ms after picture onset, similar to when and where it has been observed in previous studies on affective picture processing (cf. Cuthbert *et al.,* 2000; Moser *et al.,* 2008). In line with a large body of prior research, which found that the LPP is most pronounced over centroparietal sites (Foti and Hajcak, 2008; Wieser *et al.,* 2014), the LPP was quantified across a cluster of centroparietal electrodes (Cz, CP1, CP2, CPz, Pz; cf. Hajcak *et al.,* 2009) between 500 and 2000 ms after picture onset (cf. Hajcak and Dennis, 2009; Weinberg and Hajcak, 2010; Parvaz *et al.,* 2012; Schönfelder *et al.,* 2014).

### Statistical analysis

Affective ratings, as well as ERP measures, were subjected to separate repeated-measures ANOVAs (using SPSS version 25.0) with the within-subject factors *facial expression* (angry *vs* neutral face), *emotional intent* (escalating *vs* deescalating) and *temporal immediacy* (immediate *vs* delayed intent) and the between-subject factor *gender* to assess potential gender differences (see Syrjänen and Wiens, 2013). Effect sizes of significant results are reported as partial eta squared (*n*_p_^2^). A significance level of 0.05 (two-tailed) was used for all analyses, and *post hoc* tests were performed using Bonferroni correction. The SEM is reported for all means (mean ± SEM) if not otherwise specified.

## Results

### Affective ratings

As expected, the statistical analysis of valence ratings revealed significant main effects of *facial expression*, *F*(1,42) = 60.44, *P* < 0.001, *n*_p_^2^ = 0.590 (angry faces: 3.65 ± 0.12; neutral faces: 4.69 ± 0.09), and *emotional intent*, *F*(1,42) = 53.84, *P* < 0.001, *n*_p_^2^ = 0.562 (escalating descriptions, 3.36 ± 0.15; deescalating descriptions, 4.98 ± 0.12), although not of *temporal immediacy*, *F*(1,42) = 0.07, *P* = 0.801. Moreover, we observed significant interactions of *facial expression* and *emotional intent*, *F*(1,42) = 54.27, *P* < 0.001, *n*_p_^2^ = 0.564, and *facial expression* and *gender*, *F*(1,42) = 9.48, *P* = 0.004, *n*_p_^2^ = 0.184. Bonferroni-corrected *post hoc* tests of description categories revealed that angry faces resulted in significantly lower valence ratings than neutral faces; this difference in ratings was smaller in the escalating than in the deescalating context (escalating descriptions, *F*(1,42) = 21.64, *P (corrected)* < 0.001, *n*_p_^2^ = 0.340; deescalating descriptions, *F*(1,42) = 74.14, *P (corrected)* < 0.001, *n*_p_^2^ = 0.638).

As Levene’s test revealed that the homogeneity of variance was partially violated for both gender groups, Mann–Whitney U Tests were carried out to assess gender differences in the perception of facial expressions. The tests revealed that male participants perceived angry faces as significantly less negative compared to female participants, *U* = 129, *z* = −2.57, *P (corrected)* = 0.020.

**Fig. 2:**
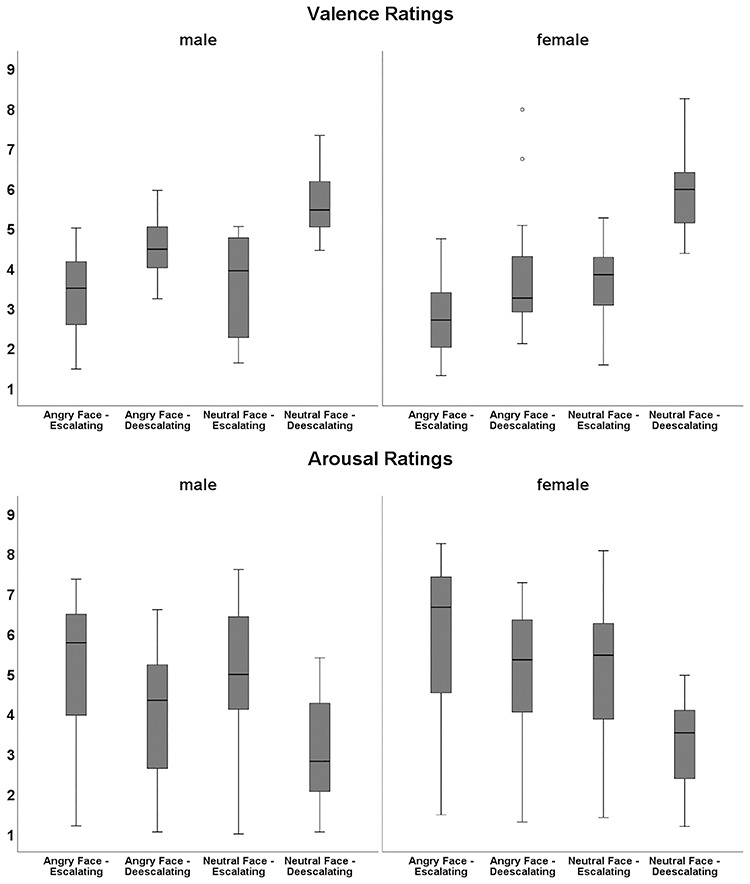
Box and whisker plots of affective ratings for angry and neutral faces preceded by deescalating and escalating descriptions. (a) Valence ratings (very negative = 1 to very positive = 9) for male and female participants. (b) Arousal ratings (not at all arousing = 1 to very arousing = 9) for male and female participants.

Significant main effects for *facial expression* and *emotional intent* were also found for arousal ratings, *F*(1,42) = 35.46, *P* < 0.001, *n*_p_^2^ = 0.458 (angry faces: 4.95 ± 0.26; neutral faces: 4.09 ± 0.20) and *F*(1,42) = 41.60, *P* < 0.001, *n*_p_^2^ = 0.498 (escalating descriptions, 5.19 ± 0.28; deescalating descriptions, 3.84 ± 0.20), respectively, whereas again no effect for *temporal immediacy* was found, *F*(1,42) = 0.49, *P* = 0.490. Similar to the valence ratings, we also found a significant interaction between *facial expression* and *emotional intent*, *F*(1,42) = 33.25, *P* < 0.001, *n*_p_^2^ = 0.442, but not for *facial expression* and *gender*, *F*(1,42) = 0.349, *P* = 0.069. Bonferroni-corrected *post hoc* tests showed that angry and neutral faces differed in perceived arousal; this difference in ratings was smaller in the escalating than in the deescalating condition (escalating descriptions, *F*(1,42) = 10.47*, P (corrected) = 0.004, n*_p_^2^ = 0.200; deescalating descriptions, *F*(1,42) = 46.74*, P (corrected) < 0.001, n*_p_^2^ = 0.527). Mean ratings per condition are depicted in Figure 2.

### The vertex positive potential

**Fig. 3:**
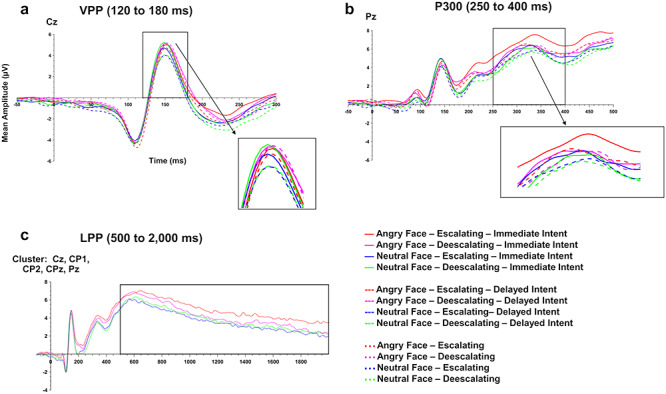
Time course of the VPP, P300 and LPP. (a) The highlighted window marks the time window of the VPP at the vertex (Cz) 120–180 ms after stimulus onset. (b) The highlighted window marks the time window of the P300 at the parietal midline (Pz) 250–400 ms after stimulus onset. (c) Time course of the LPP at a centroparietal cluster (Cz, CP1, CP2, CPz, Pz) 500–2000 ms after stimulus onset.

**Fig. 4:**
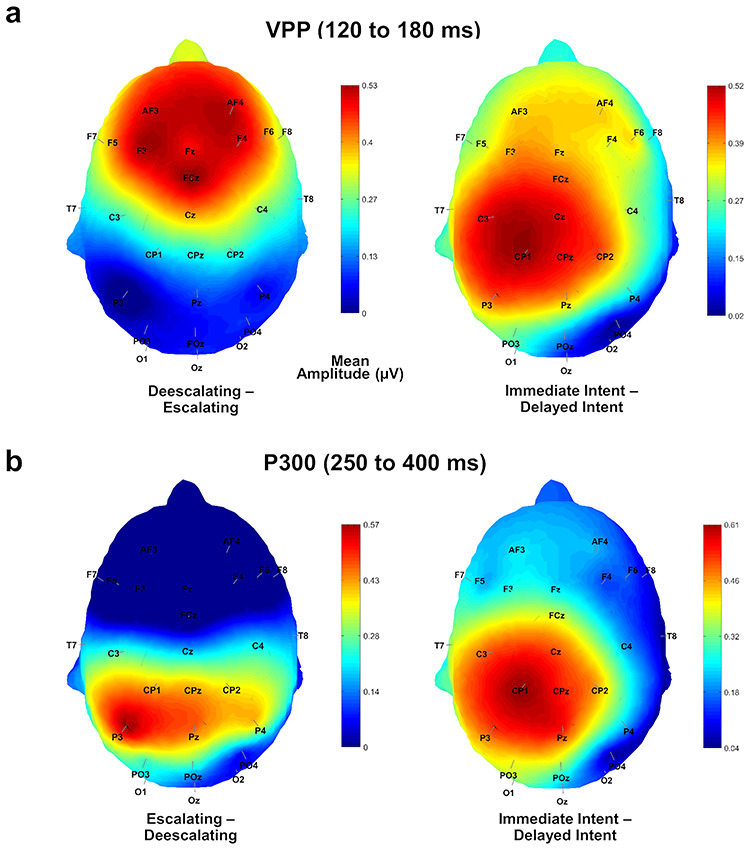
Topographic differences maps of the VPP and P300. (a) Topographic difference maps of mean activity in the VPP time interval (120–180 ms) after stimulus onset. Left: mean difference for deescalating and escalating descriptions. Right: mean difference for descriptions signaling an immediate or delayed intent. (b) Topographic difference maps of mean activity in the P300 time interval (250–400 ms) after stimulus onset. Left: mean difference for deescalating and escalating descriptions. Right: mean difference for descriptions signaling an immediate or delayed intent.

The mean amplitude of the VPP at the vertex (Cz) differed significantly for *facial expression*, *F*(1,40) = 4.75, *P* = 0.035, *n*_p_^2^ = 0.106 (angry faces: 2.47 ± 0.66; neutral faces: 2.09 ± 0.66); *emotional intent*, *F*(1,40) = 6.90, *P* = 0.012, *n*_p_^2^ = 0.147 (escalating descriptions: 2.08 ± 0.64; deescalating descriptions: 2.48 ± 0.67); and *temporal immediacy*, *F*(1,40) = 15.66, *P* < 0.001, *n*_p_^2^ = 0.281 (immediate intent: 2.50 ± 0.64; delayed intent: 2.06 ± 0.66; see Figures 3 and 4). No interaction effects between the different factors, or gender effects, were found, all *F*s < 2.56, *Ps* > 0.117.

### The P300

Similar to the VPP, the analysis of the P300 at Pz revealed significant main effects of *facial expression*, *F*(1,40) = 17.09, *P* < 0.001, *n*_p_^2^ = 0.299 (angry faces: 5.78 ± 0.69; neutral faces: 4.98 ± 0.64); *emotional intent*, *F*(1,40) = 6.32, *P* = 0.016, *n*_p_^2^ = 0.136 (escalating descriptions, 5.60 ± 0.68; deescalating descriptions: 5.16 ± 0.65); and *temporal immediacy*, *F*(1,40) = 9.12, *P* = 0.004, *n*_p_^2^ = 0.186 (immediate intent: 5.65 ± 0.67; delayed intent: 5.10 ± 0.66; see Figures 3 and 4). No interaction effects were found for any of the factors, or for gender, all *F*s < 3.40, *P*s > 0.072.

### The late positive potential

The analysis of the LPP at the cluster of centroparietal electrodes (Cz, CP1, CP2, CPz, Pz) revealed significant effects for *facial expression*, *F*(1,40) = 7.41, *P* = 0.010, *n*_p_^2^ = 0.156 (angry faces, 4.60 ± 0.69; neutral faces, 3.73 ± 0.67), but no effect for *emotional intent*, *F*(1,40) = 0.53, *P* = 0.472, or *temporal immediacy*, *F*(1,40) = 1.82, *P* = 0.185 (see Figure 3). Moreover, we found a significant interaction for *facial expression* and *emotional intent*, *F*(1,40) = 4.44, *P* = 0.041, *n*_p_^2^ = 0.100. Bonferroni-corrected *post hoc* tests showed that the LPP amplitude to angry compared to neutral faces was significantly different when preceded by escalating descriptions (*F*(1,40) = 10.42, *P (corrected)* = 0.004, *n*_p_^2^ = 0.207), whereas no such difference could be observed for the LPP amplitude to any faces preceded by deescalating descriptions (*F*(1,40) = 1.15, *P (corrected)* = 0.580, *n*_p_^2^ = 0.028; angry faces/escalating descriptions: 4.96 ± 0.77; angry faces/deescalating descriptions: 4.25 ± 0.67; neutral faces/escalating descriptions: 3.60 ± 0.66; neutral faces/deescalating descriptions: 3.85 ± 0.73). No interactions between any of the other factors, or gender, reached significance, all *F*s < 2.96, *P*s > 0.93.

## Discussion

The present study aimed to investigate how threat- and time-related information, conveyed by contextual descriptions, affected the processing of facial stimuli. To this end, participants viewed angry and neutral faces preceded by descriptions indicating *emotional intent* (escalating *vs* deescalating) and *temporal immediacy* (immediate intent *vs* delayed intent) while we recorded their scalp EEG and obtained affective ratings.

In line with previous research and as expected, angry (threatening) faces were associated with significantly increased amplitudes for both early (VPP) and late (P300, LPP) ERP components (cf. Schupp *et al.,* 2004; Foti *et al.,* 2010; Duval *et al.,* 2013; Smith *et al.,* 2013). Moreover, we found independent effects of *emotional intent* and *temporal immediacy* on the VPP and P300 while an interaction of *emotional intent* with *facial expression* emerged during the LPP time interval. We also found an interaction of *emotional intent* and *facial expression* for valence and arousal ratings, but no effect of *temporal immediacy*.

Faces preceded by descriptions signaling immediate intent resulted in a significantly enhanced VPP amplitude compared to descriptions signaling delayed intent, irrespective of *facial expression* or the *emotional intent* of the descriptions. This finding is in line with prior research that shows that contextual influences to faces or visual scenes may emerge already in the VPP/N170 time window (Conty *et al.,* 2007, 2012; Righart and De Gelder, 2008; Hirschfeld *et al.,* 2012; Diéguez-Risco *et al.,* 2015). The analysis of the P300 revealed that the effect of *temporal immediacy* on the processing of facial expressions persisted during the P300 time interval: consistent with studies on delay discounting, immediate-intent descriptions were associated with a significantly larger P300 amplitude than delayed-intent descriptions (Gui *et al.,* 2016), indicating that temporal immediacy enhances the motivational relevance of a stimulus. As the contextual information was presented prior to the faces, we believe that these early independent effects of *temporal immediacy* may reflect an increased pre-allocation of attentional resources to the ensuing facial expressions, presumably to expedite the preparation of an adaptive response (cf. Conty *et al.,* 2012).

Moreover, we observed an independent effect of *emotional intent* on the VPP as well as on the P300. Surprisingly, and in contrast to our original predictions, deescalating descriptions resulted in a higher VPP amplitude than escalating descriptions, although they showed the reverse (and expected) effect for the P300. A potential explanation for the observed effect of *emotional intent* on the VPP could be that the processing of faces preceded by deescalating descriptions required a larger cognitive effort, possibly because of its more complex integration of context and facial expression. In contrast to escalating descriptions (e.g. ‘She/he is about to hit somebody’), deescalating descriptions were arguably more equivocal in terms of their affective valence, as they referred implicitly to interpersonal discord in the past and explicitly to the resolution of the discord in the immediate or distant future (e.g. ‘She/he is about to forgive somebody’). Facial expressions following deescalating descriptions could therefore have required a double evaluation of the valence, since neither neutral nor angry faces reflected the expectations engendered by the deescalating descriptions (see Aguado *et al.,* 2018, 2019 for a discussion of congruency effects between affective contextual descriptions and facial expressions). Support for this explanation that the relatively higher complexity of deescalating compared to escalating descriptions modulated the VPP amplitude comes from studies that found picture complexity to alter early perceptual processes, but not later components such as the P300 or LPP (Bradley *et al.,* 2007). However, this explanation remains speculative, and future research is needed to investigate the roles that affective ambiguity and complexity of contextual information might play in the early processing of facial expressions.

Interestingly, an interaction between *facial expression* and *emotional intent* emerged during the LPP time window: angry faces in an escalating context resulted in a larger LPP amplitude than neutral faces in an escalating context. In contrast, a deescalating context eliminated LPP differences between angry and neutral faces. This finding corresponds only in part to the observed modulation of affective ratings by facial expression and emotional intent. While the enhanced difference in LPP amplitude between angry and neutral faces in an escalating context is consistent with an increased difference in valence and arousal ratings, no coherent effect of LPP measures and affective ratings could be found for faces presented in a deescalating context. Contrary to the LPP measures, valence and arousal ratings differed for angry and neutral faces following deescalating descriptions.

Notably, a similar pattern of results for the LPP was reported by Wessing *et al.* (2013) in a magnetoencephalography study on the neural basis of reappraisal, in which participants were instructed to either attentively view, down or upregulate their emotional response to angry and neutral faces. In line with our findings, Wessing *et al.* (2013) found an enhanced LPP amplitude for angry compared to neutral faces during upregulation but no significant difference in LPP amplitude between angry and neutral faces in their downregulation condition. The authors attributed this to a potential floor effect, i.e. that angry faces were not perceived as threatening enough to be further reduced via downregulation. Similarly, the level of threat evoked by angry faces in our study may have been insufficient to elicit a pronounced LPP amplitude, thereby limiting our potential to probe the modulatory influence of facial expression in a deescalating context. Note, however, that this explanation cannot fully account for the discrepancy between LPP measures and affective ratings in the deescalating context. We discuss potential reasons for this inconsistency further below.

Alternatively, it is possible that neutral faces in combination with the deescalating context were perceived as positive rather than neutral stimuli. As both positive and negative stimuli have been shown to increase the LPP amplitude (Olofsson *et al.,* 2008), this could have potentially reduced an effect between (positively perceived) neutral and (negatively perceived) angry faces. This explanation is supported by valence ratings that were on average positive for neutral faces (i.e. ratings >5) and negative for angry faces (i.e. ratings <5) in the deescalating condition (see Supplementary Materials for one-sample *t*-test statistics), but cannot account for the pattern observed for arousal ratings: as the LPP amplitude is also sensitive to stimulus arousal levels (Rozenkrants *et al.,* 2008), lower arousal ratings for neutral than for angry faces in the deescalating condition would have suggested a reduced LPP amplitude for neutral compared to angry faces.

Importantly, differences in self-report data and neural activity could also reflect a dissociation between early perceptual processing stages (as evidenced by ERPs) and conscious evaluation processes (behavioral ratings) (Mühlberger *et al.,* 2009). Other variance-contributing factors may be intermediary variables, such as the participant’s interoceptive or emotional awareness as well as their capability to accurately translate emotions into numerical reports (MacNamara *et al.,* 2009).

Moreover, participants were only asked to rate facial stimuli (and not the preceding descriptions) in terms of valence and arousal. This could also explain why we observed a specific effect of *temporal immediacy* that deescalating descriptions with immediate intent were rated as specific effect of temporal immediacy, i.e. deescalating descriptions with delayed intent in the online pilot study (see Supplementary Materials), but not in the ratings obtained in the present study.

### Limitations

One potential limitation of our study was that both description types referred to interpersonal discord. Although we selected descriptions based on the arousal ratings obtained in a pilot study, such that escalating descriptions were rated as most arousing and deescalating descriptions as least arousing, while maintaining a negative or positive valence, respectively, the design of the study did not allow us to determine the directional influence of contextual effects on neural responses as we had no neutral control condition. Moreover, we obtained valence and arousal ratings for each facial expression, which may have influenced the processing of the faces (see Aguado *et al.,* 2019; Van Strien *et al.,* 2010, for a discussion).

### Conclusion

The present results are in line with a growing body of studies that support the view that contextual (top-down) information influences the perception of facial expressions on both the behavioral and cortical level. Importantly, our findings demonstrate, for the first time, that in addition to contextual affective content, other factors, such as temporal immediacy, may influence early stages of facial processing, as evidenced by increased VPP and P300 amplitudes to faces preceded by descriptions signaling immediate intent. The observed independent effects of emotional intent and temporal immediacy underline the need for a systematic assessment of situational variables in context–face studies. The extent to which temporal immediacy modulates emotional reactivity to facial stimuli is yet to be fully explored, though our results suggest that temporal distancing may be an effective means of modulating emotional arousal in such contexts.

## Conflicts of interest

The authors declare that no conflicts of interest exist.

## Supplementary Material

scan-19-098-File008_nsaa071
