# Supplementary material for: In context: emotional intent and temporal immediacy of contextual descriptions modulate affective ERP components to facial expressions
Source: Soc Cogn Affect Neurosci. 2020 May 21;15(5):551–60. doi: 10.1093/scan/nsaa071 (PMC7328032; doi:10.1093/scan/nsaa071)
Supplement: scan-19-098-File008_nsaa071 [file scan-19-098-file008_nsaa071.docx]

**Supplementary Materials**

*Results of pre-experimental online questionnaire ratings for 64 selected descriptions*

A repeated-measures ANOVA with the within-subject factors *emotional intent* (escalating vs. deescalating) and *temporal immediacy* (immediate intent vs. delayed intent) revealed that ratings differed significantly with regards to *emotional intent* for both valence and arousal ratings, *F*(1,17) = 190.13, *p* < .001, *ɳ_p_^2^* = .918, and *F*(1,17) = 139.64, *p* < .001, *ɳ_p_^2^* = .891, respectively. A significant main effect for *temporal immediacy* could be observed for arousal ratings *F*(1, 17) = 6.00, *p* < .025, *ɳ_p_^2^* = .261, and a significant interaction effect for valence ratings, *F*(1,17) = 7.91, *p* = .012, *ɳ_p_^2^* = .318. Bonferroni-corrected post-hoc comparisons showed that deescalating descriptions with immediate intent resulted in higher valence ratings than did deescalating descriptions with delayed intent, whereas no differences were observed in the escalating condition, *F*(1,17) = 9.208, *p* (*corrected*) = .014, *ɳ_p_^2^* = .351 (see Table 1).

Note that we used the gender-neutral Swedish pronoun "hen" for all descriptions in the online pilot questionnaire.

**Table 1.**

Mean affective ratings ±SD (valence and arousal) of descriptions varying in *emotional intent* and *temporal immediacy* in a pilot study.

|  |  | **Temporal immediacy** |  |  |
| --- | --- | --- | --- | --- |
|  | **Immediate intent** |  | **Delayed intent** |  |
| *Emotional intent* | Valence | Arousal | Valence | Arousal |
| *Deescalating* | 6.94 (1.08) | 3.42 (1.60) | 6.66 (1.12) | 3.34 (1.55) |
| *Escalating* | 1.69 (.58) | 7.55 (1.13) | 1.80 (.75) | 7.18 (1.61) |

*Perceived valence of angry and neutral faces in the deescalating condition*

To determine whether neutral faces following a description signaling a deescalating intent were perceived as positive, we run a one-sample (2-tailed) t-test comparing ratings against a test value of 5 (i.e. the neutral point on the 9-point Likert scale). Results revealed that valence ratings were significantly higher than 5 (*M* = 5.74, *SD* = .86), suggesting that neutral faces in the deescalating condition were perceived as positive, *t*(43) = 5.59, *p* < .001. In contrast, valence ratings for angry faces in the deescalating condition were significantly lower than 5 (*M* = 4.26, *SD* = 1.19), suggesting that angry faces were perceived as negative, *t*(43) = -4.14, *p* < .001.

*32 descriptions preceding angry and neutral faces*

**Table 2.** The 16 deescalating and 16 escalating descriptions selected for the study. Note that all descriptions signaled an immediate intent (which can be roughly translated with “is about to do something”) as well as a delayed intent (which can be roughly translated with “is going to do something later”).

| **Immediate intent –**  **Deescalating description** | **Delayed intent –**  **Deescalating description** | **English translation** |
| --- | --- | --- |
| Hen är på väg att gottgöra någon | Hen kommer att gottgöra någon senare | She/he will make it up to someone |
| Hen är på väg att försonas med någon | Hen kommer att försonas med någon senare | She/he will reconcile with someone |
| Hen är på väg att godta någons ursäkt | Hen kommer att godta någons ursäkt senare | She/he will accept someone’s apology |
| Hen är på väg att sluta fred med någon | Hen kommer att sluta fred med någon senare | She/he will make peace with someone |
| Hen är på väg att komma överens med någon | Hen kommer att komma överens med någon senare | She/he will agree with someone |
| Hen är på väg att lösa en dispyt med någon | Hen kommer att lösa en dispyt med någon senare | She/he will resolve a dispute with someone |
| Hen är på väg att förlåta någon | Hen kommer att förlåta någon senare | She/he will forgive someone |
| Hen är på väg att sluta gräla med någon | Hen kommer att sluta gräla med någon senare | She/he will stop arguing with someone |
| Hen är på väg att komma till rätta med någon | Hen kommer att komma till rätta med någon senare | She/he will make amends to someone |
| Hen är på väg att förstå någons problem | Hen kommer att förstå någons problem senare | She/he will understand someone’s problem |
| Hen är på väg att lita på någon | Hen kommer att lita på någon senare | She/he will trust someone |
| Hen är på väg att godta någons brister | Hen kommer att godta någons brister senare | She/he will accept someone’s flaws |
| Hen är på väg att undvika en konflikt med någon | Hen kommer att undvika en konflikt med någon senare | She/he will avoid a conflict with someone |
| Hen är på väg att acceptera någon | Hen kommer att acceptera någon senare | She/he will accept someone |
| Hen är på väg att kompromissa med någon | Hen kommer att kompromissa med någon senare | She/he will compromise with someone |
| Hen är på väg att be om ursäkt till någon | Hen kommer att be om ursäkt till någon senare | She/he will apologize to someone |
| **Immediate intent –**  **Escalating description** | **Delayed intent –**  **Escalating description** | **English translation** |
| Hen är på väg att kränka någons rättigheter | Hen kommer att kränka någons rättigheter senare | She/he will violate someone’s rights |
| Hen är på väg att mobba någon | Hen kommer att mobba någon senare | She/he will bully someone |
| Hen är på väg att trakassera någon | Hen kommer att trakassera någon senare | She/he will harass someone |
| Hen är på väg att överfalla någon | Hen kommer att överfalla någon senare | She/he will assault someone |
| Hen är på väg att ge någon stryk | Hen kommer att ge någon stryk senare | She/he will beat someone up |
| Hen är på väg att slå ner någon | Hen kommer att slå ner någon senare | She/he will punch someone |
| Hen är på väg att förlöjliga någon | Hen kommer att förlöjliga någon senare | She/he will ridicule someone |
| Hen är på väg att råna någon | Hen kommer att råna någon senare | She/he will mug someone |
| Hen är på väg att misshandla någon | Hen kommer att misshandla någon senare | She/he will abuse someone |
| Hen är på väg att skada någon | Hen kommer att skada någon senare | She/he will hurt someone |
| Hen är på väg att sparka någon | Hen kommer att sparka någon senare | She/he will kick someone |
| Hen är på väg att attackera någon | Hen kommer att attackera någon senare | She/he will attack someone |
| Hen är på väg att strypa någon | Hen kommer att strypa någon senare | She/he will strangle someone |
| Hen är på väg att bryta näsan på någon | Hen kommer att bryta näsan på någon senare | She/he will break someone’s nose |
| Hen är på väg att spotta på någon | Hen kommer att spotta på någon senare | She/he will spit on someone |
| Hen är på väg att slå någon | Hen kommer att slå någon senare | She/he will hit someone |
